# The burnout enigma solved?

**DOI:** 10.5271/sjweh.3950

**Published:** 2021-03-31

**Authors:** Wilmar Schaufeli

**Affiliations:** Professor Emeritus of Work and Organizational Psychology Social, Health & Organizational Psychology, Utrecht University, The Netherlands

In the current issue of the *Scandinavian Journal of Work, Environment and Health*, an international expert panel presented a consensual definition on burnout that reads as follows: “*In a worker, occupational burnout* or *occupational physical AND emotional exhaustion state* *is an exhaustion due to prolonged exposure to work-related problems*.” (1, p95) Does this definition solve the burnout enigma? Obviously, the quest for an agreed-upon definition has haunted burnout research from the moment the concept was introduced by Freudenberger in the mid 1970s (2). Needless to say, the absence of consensus on what burnout ‘is’ has hampered the accumulation of our knowledge on its epidemiology, antecedents, and consequences and interventions to reduce it (3).

But is it really true that scholars completely disagree about burnout – as the panel assumes? The answer to this question is affirmative as well as negative, just like quantum particles that can be charged both positively and negatively at the same time. On the one hand, virtually all scholars agree that exhaustion is the core, constituting component of burnout. Tellingly, exhaustion is mentioned in 12 of the 13 definitions that are listed in Table 1 of the panel’s paper. Also, the Maslach Burnout Inventory (MBI), which prominently includes an exhaustion subscale, is used almost universally in about 90% of all studies on burnout (4). On the other hand, it is not clear what *kind* of exhaustion is supposed to be typical for burnout: emotional, physical, mental, or cognitive exhaustion? Or all of these? And how do they differ, for that matter? To the best of my knowledge the nature and the different kinds of exhaustion have not been discussed systematically in the burnout literature so far. In addition, although most scholars would agree that exhaustion is a necessary condition for burnout, they disagree on whether it is a sufficient condition as well. For instance, for some, burnout is equivalent to exhaustion, and they argue that the two other dimensions that are included in the MBI are unnecessary as (i) mental distancing (ie, cynicism or depersonalization) refers to a way of coping and (ii) reduced personal accomplishment is a consequence of burnout (5). In contrast, for others such as myself, exhaustion is a necessary yet insufficient component for burnout as will be explained below.

The panel chose a pragmatic way of cutting the Gordian burnout knot by looking for the common denominator in the definitions used so far. Not surprising – and in fact quite predictably – work-related (emotional and physical) exhaustion appeared on the scene. In my opinion, the definition of burnout of the panel is problematic for three reasons. First, the nature of work is not specified. It seems that the definition is restricted to formal employment relationships as the exhaustion is termed as ’occupational’. That would mean that – quite incongruously – the first burnout cases Freudenberger described, who happened to be *volunteers* working with drug addicts, would not have suffered from the syndrome. And what about students, athletes and parents, for instance? Can they burn out? According to the panel’s definition they cannot, despite numerous papers on the subject. Or does the adjective ‘occupational’ in the definition imply that there are other non-occupational forms of burnout as well? Be as it may, should we abandon the work-related nature of burnout altogether? Not quite, if we understand work psychologically and not in terms of labor relations – that is when all structured, goal-oriented activities that are compulsory in nature and aim to transform the physical or social environment and/or the self are considered ‘work’. Seen from that psychological perspective, volunteers, students, athletes and parents do ‘work’ because they are engaged in such activities.

Second, in the definition burnout is equated with a combination of emotional and physical exhaustion: but what about the other kinds of exhaustion? An what exactly is emotional exhaustion? Is it different from mental exhaustion? Does the “… *exhaustion* *due to prolonged exposure to work-related problems”* include cognitive exhaustion as well, which manifests itself in typical burnout complaints such as lack of concentration and inability to think clearly? Unfortunately, these questions remain unanswered. In short, the exact nature of exhaustion and the domain in which it reveals itself remains unclear.

Last but not least, because of the pragmatic approach of the panel, their burnout definition lacks a proper theoretical underpinning. Essentially, exhaustion is a state of being extremely fatigued. A long tradition exists on work-related fatigue beginning with the seminal work of Edward Thorndike (1874–1949), who maintained that the basic tenet of fatigue is “the intolerance of any effort” (6, p104). In his view, fatigue is both the inability *and* the unwillingness to spend effort at work, which is reflected by its energetic and motivational component, respectively. The unwillingness to perform is expressed in increased resistance, reduced commitment, cynicism, lack of interest, disengagement, and so on – in short, mental distancing. Building on the work of Thorndike, Schaufeli & Taris (7) have argued that inability (exhaustion) and unwillingness (distancing) constitute two sides of the same burnout coin. Hence, based on a theoretical analysis mental distancing should be recognized in addition to exhaustion as the second constituting dimension of burnout (8). This meshes with the results of a recent review of 12 burnout questionnaires, 8 of which assessed burnout as a multidimensional concept; *all* of which included an exhaustion as well as a mental distance subscale (the remaining 3 questionnaires reduced burnout to mere exhaustion) (8).

So, taken together,it seems that – notwithstanding the panel’s laudable endeavor – the burnout enigma has not yet been solved. This means that, despite the agreement about exhaustion as a key element of burnout, the discussion about the definition of burnout continues.
